# A Case of Severe Carotid Stenosis in a Patient with Familial Hypercholesterolemia without Significant Coronary Artery Disease

**DOI:** 10.1155/2014/853921

**Published:** 2014-10-23

**Authors:** Marcos Aurélio Lima Barros, Hygor Ferreira-Fernandes, Ingrid Cristina Rêgo Barros, Adriel Rêgo Barbosa, Giovanny Rebouças Pinto

**Affiliations:** ^1^Marcor Clinic, Maximum Body Care and Recovery, Avenida Presidente Vargas 811, 64200-200 Parnaíba, PI, Brazil; ^2^Genetics and Molecular Biology Laboratory, Federal University of Piauí, Avenida São Sebastião 2819, 64202-020 Parnaíba, PI, Brazil; ^3^Faculty of Medicine, Federal University of Piauí, Avenida São Sebastião 2819, 64202-020 Parnaíba, PI, Brazil; ^4^Faculty of Medicine, Federal University of Piauí, R. Doutor Natan Portela Nunes s/n, 64049-550 Teresina, PI, Brazil

## Abstract

Familial hypercholesterolemia (FH) is an inherited metabolic disorder characterized by elevated low-density lipoprotein cholesterol levels in the blood. In its heterozygous form, it occurs in 1 in 500 individuals in the general population. It is an important contributor to the early onset of coronary artery disease (CAD), accounting for 5–10% of cases of cardiovascular events in people younger than 50 years. Atherogenesis triggered by hypercholesterolemia generally progresses faster in the coronary arteries, followed by the subsequent involvement of other arteries such as the carotids. Thus, symptoms of CAD commonly appear before the onset of significant carotid stenosis. Herein, we report the case of a patient with untreated FH who had severe carotid atherosclerosis at the age of 46 years but had no evidence of significant CAD.

## 1. Introduction

Coronary artery disease (CAD) belongs to the spectrum of cardiovascular diseases, which are the leading cause of death worldwide [1]. Atherosclerosis of the coronary arteries is the main cause of CAD [2], which is linked to various risk factors [1]. High cholesterol levels in the blood, caused by high-fat diets, contribute significantly to the onset and progression of atherosclerosis of the coronary arteries [1]. However, in some conditions, even with a healthy diet, cholesterol levels remain constantly elevated, dramatically increasing the risk of CAD. This is the case in familial hypercholesterolemia (FH), an inherited metabolic disorder characterized by remarkably high levels of low-density lipoprotein cholesterol (LDL-C) due to genetic mutations that reduce the number or impair the function of the LDL receptors on the surface of hepatocytes. Thus, cholesterol massively accumulates in tissues, causing characteristic clinical signs such as tendon xanthomas and increasing the risk for premature CAD [3].

FH is a serious disease, with an incidence of 1 in 500 individuals in its heterozygous form, accounting for 5–10% of cases of cardiovascular events, mainly in people younger than 50 years [4]. When untreated, up to 85% of individuals with heterozygous FH will have a coronary event before the age of 65 years, of whom approximately 50% survive beyond the age of 60 years [5]. Another consequence of high cholesterol levels in patients with FH is carotid artery stenosis, increments of which increase the risk for complications, depending on the degree of obstruction and instability of plates [6]. Despite its systemic nature, atherosclerotic disease does not uniformly affect the different vascular territories. Many studies point to carotid artery stenosis as a predictor of the presence of CAD, indicating that atherogenesis progresses faster in coronary arteries than in carotids, meaning that symptoms of CAD commonly appear before the onset of significant carotid stenosis [7–9]. Here we report the case of a patient diagnosed with FH who presented with severe atherosclerosis of the carotid arteries but no evidence of significant CAD.

## 2. Case Report

**Figure 1:**
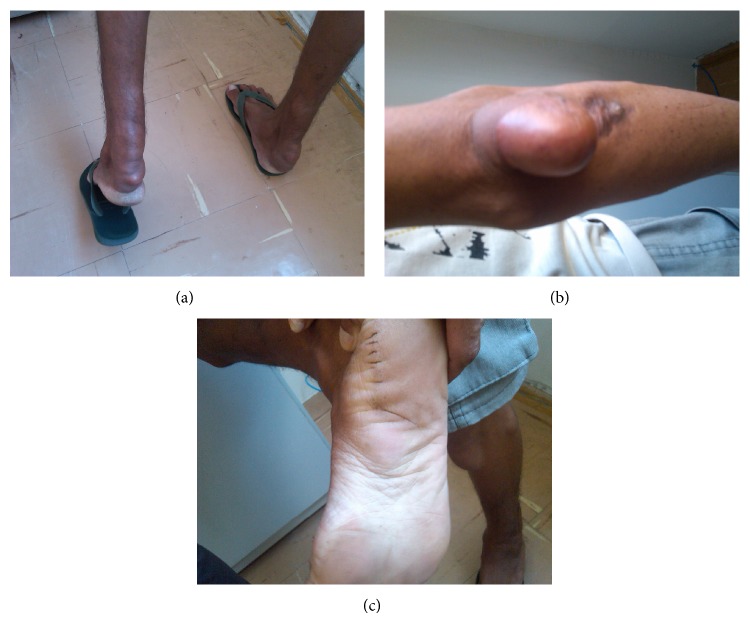
Clinical presentation of the patient. Xanthomas on the heels (a), elbows (b), and soles of the feet (c).

A 46-year-old man (weight, 78 kg; height, 172 cm), who resided in the countryside of Parnaíba, Piauí, Brazil, consulted us for complaints of dizziness and frequent headaches. He was diagnosed with FH according to the clinical and laboratory criteria established in the Brazilian Guideline for Familial Hypercholesterolemia (based on the criteria of the Dutch Lipid Clinic Network) [4]. In addition, he had xanthomas on the elbows, soles of the feet, and Achilles tendons (Figure 1) and a family history of CAD, since his father died from acute myocardial infarction (the patient was unable to inform the occurrence of CAD in other family members). The patient had no other cardiovascular risk factors such as smoking, high blood pressure, and diabetes mellitus and was physically active. The patient's LDL-C and HDL-C levels were 545 and 53 mg/dL, respectively. His fasting glucose and triglyceride levels (85 and 158 mg/dL, resp.) were within normal limits.

The patient reported no use of lipid-lowering medication until the time of diagnosis. For socioeconomic reasons and/or lack of instruction, he had not sought medical care until when his symptoms eventually made him unable to work in the field. However, no symptoms of CAD were revealed.

**Figure 2:**
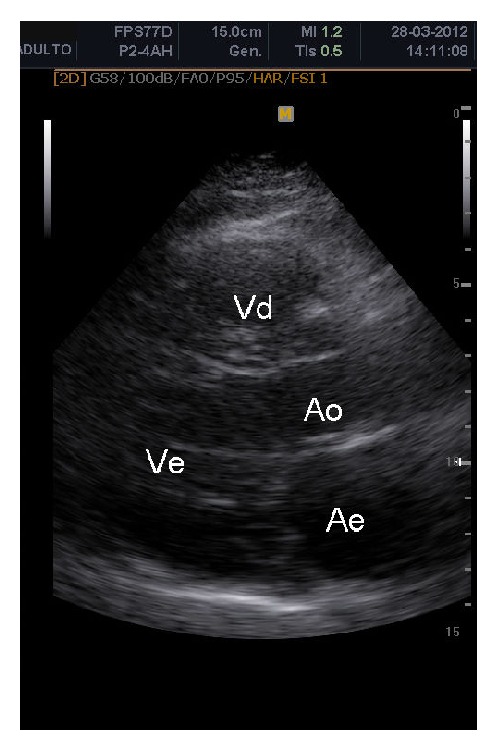
Visualization of the aortic root and aortic valve, both without signs of involvement.

**Figure 3:**
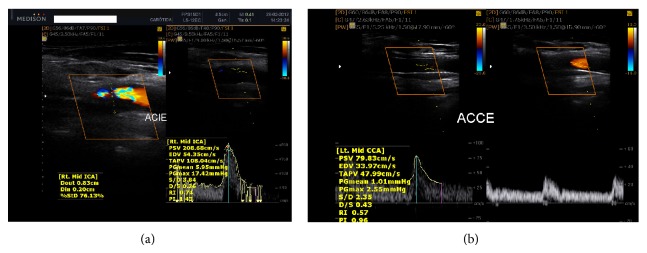
Ultrasonography of the carotid arteries showing the presence of plaques in the left internal carotid artery (a) and left internal carotid artery after stent implantation (b).

Neck auscultation revealed a systolic murmur 3+/6+ in the neck, radiating to the skull. Electrocardiography, chest radiography, stress testing, Holter monitoring, and echocardiography revealed no abnormalities. Visual examination of the aortic root and aortic valve revealed no signs of their involvement (Figure 2). On ultrasonography of the carotid arteries, we observed severe stenosis in the left internal carotid artery (LICA), with stenosis estimated between 70% and 90% (according to the criteria established by the European Carotid Surgery Trial and North American Symptomatic Carotid Endarterectomy Trial, as well as Dopplervelocimetric data) and moderate stenosis in the right internal carotid artery (RICA) estimated between 40% and 50% (Figure 3(a)). For the LICA, the peak-systolic (PSV) and end-diastolic velocity (EDV) cutoff values were 208.5 cm/s and 54.5 cm/s, respectively (Dex: 0.83 cm; Dint: 0.20 cm; %Std: 76.13); RICA PSV was 91.72 cm/s and RICA EDV was 37.37 cm/s (Dex: 0.74 cm; Dint: 0.44 cm; %Std: 41.27). We observed the presence of plaques in the anterior and posterior walls of the internal carotid artery and common carotid artery, which were characterized as bulky plates extending to the middle third of the internal coronary arteries (ICAs) and as predominantly echogenic and hyperechoic, with less than 50% of the area being echolucent with uneven surfaces, according to the Classification of Atherosclerotic Carotid Boards [10].

A low-cholesterol diet was immediately recommended to the patient, and clinical treatment with statins was initiated. Concurrently, the patient underwent percutaneous revascularization by stent placement in the common and left internal carotid artery, and postoperative control showed adequate blood flow (Figure 3(b)). No intraoperative or postoperative complications were encountered. The patient remained asymptomatic, but the LDL-C levels remained high (320 mg/dL).

## 3. Discussion

FH is recognized by the World Health Organization as a global public health problem. However, the failure of health systems to identify FH early enough obscures the true incidence of the disease and thus prevents the delivery of proper treatment to patients. The worldwide estimate of the number of individuals with FH is more than 10 million; however, fewer than 10% of these individuals have known diagnoses and fewer than 25% receive treatment for hyperlipidemia [11]. Making this situation even more alarming is the high incidence of premature atherosclerosis in these individuals, which reduces their life expectancy [4].

The case presented in this paper represents an additional example of delayed FH diagnosis, wherein the patient was exposed to the consequences of significant hypercholesterolemia, without any lipid-lowering therapy, until the age of 46 years. Despite the clear clinical signs such as xanthomas on the elbows, soles of the feet, and Achilles tendons, the patient had not sought appropriate medical care. He also did not have any serious symptoms such as those related to CAD for a long time; the carotid stenosis, manifested through headache and dizziness, and occupational difficulty motivated him to eventually consult us.

Ultrasonography of the carotid arteries revealed severe stenosis in the LICA (70–90%) and moderate stenosis in the RICA (40–50%). Meanwhile, the cardiac evaluation test results were normal; the aorta showed no atherosclerotic changes. In addition, the patient's medical history showed no signs of significant obstruction in the coronary arteries. This is an unusual fact considering that the presence of severe carotid stenosis without apparent coronary and aortic involvement is uncommon in clinical practice. Especially in adult patients with untreated FH, the constantly elevated LDL-C levels should accelerate the process of atherosclerosis, which in such patients initially affects the aortic root, extending to the coronary ostium [12].

The atherosclerotic process presents a generalized character, occurring simultaneously in different vascular territories. Therefore, it is reasonable to infer that patients with severe atherosclerosis in certain arterial territories are more likely to present significantly the characteristic in the remaining territories [13]. The existing literature clearly shows the coexistence of carotid artery atherosclerosis with arterial vascular disease in other branches, with CAD being the most important [14]. In the study by Paraskevas et al. [14] that included 120 patients with ICA occlusion, 70% of the patients had concomitant vascular disease in other arteries, with 78.5% corresponding to CAD disease.

However, despite being a systemic disease, atherosclerosis manifests its symptoms in a focal manner. Vascular disturbances in blood flow favor the development of lesions, primarily in certain environments. Strong evidence suggests that the significant involvement of the coronary arteries usually precedes carotid artery stenosis, indicating that the onset and progression of the atherosclerotic process are slower in the latter. Kablak-Ziembicka et al. [15] evaluated 558 patients with suspected CAD who underwent coronary angiography and carotid ultrasonography and found that none of the patients with normal coronary arteries had severe extracranial artery stenosis. Conversely, severe carotid artery stenosis was found in 16.6% of the patients with three-vessel CAD. This strong relationship between carotid artery stenosis and CAD has warranted the use of the measurement of the carotid intima-media thickness (CIMT) as a noninvasive indicator of the atherosclerotic process in the coronary arteries. Such measurement was reported to have 100% sensitivity and 50% specificity in detecting significant CAD [16], although the addition of CIMT to traditional cardiovascular risk prediction models does not lead to a statistically significant increase in performance of those models [17].

Patients with FH are exposed to the risk of hypercholesterolemia since childhood. Without drug therapy and without taking into account the influence of other risk factors on cholesterol levels, this risk factor remains constant throughout the life of these patients. These patients exhibit more advanced atherosclerosis and more severe organic disorders than patients with typical hypercholesterolemia, without a relevant genetic background. In this case, the relationship between CAD and carotid stenosis has become even more evident. The study by ten Kate et al. [18] exemplifies this close relationship. The authors found that the absence of carotid plaques observed in 5 patients with heterozygous FH (7%) excludes the presence of obstructive CAD. The idea that the significant involvement of the carotid artery precedes that of the coronary artery is highlighted in this report. Our case has drawn attention due to the fact that atherosclerotic lesions were not observed in the aorta, this being the primary site of lesions in patients with FH [12]. Caballero et al. [19] observed that all patients with plaques in carotid arteries (5/36) also had plaques in the aorta and, on the other hand, the two FH cases without plaques in the aorta had no plaques in carotid arteries.

Palacio et al. [20] described the case of a 3-year-old girl diagnosed with homozygous FH, which was confirmed by mutational analysis of the LDL receptor gene. The girl showed similar clinical characteristics as those of our patient, that is, a normal aortic valve without aortic stenosis, no calcification, and normal coronary arteries with no evidence of stenosis or calcification despite carotid intimal thickening and plaques. In our case, a genetic analysis could not be performed because the test was unavailable. In patients with untreated homozygous FH, the rapid progression of atherosclerotic changes, culminating especially in aortic stenosis and CAD, is the usual cause of death before the age of 20 years [21]. Heterozygous FH is more frequently associated with cardiovascular complications in middle-aged patients [22]. Our patient had no episode of ischemic heart disease by the age of 46 years, suggesting a case of heterozygous FH.

The clinical presentation of CAD in patients with FH is heterogeneous in terms of age of onset and severity [23]. However, highlighted in this report is not the absence of CAD in an adult patient with untreated FH but the presence of severe carotid stenosis with no evidence of significant CAD. Stent placement in the obstructed has carotid successfully restored the cerebrovascular blood flow of the patient. The patient's complaints of dizziness and headache were resolved after the procedure.
